# Identification of Immunoglobulin Gene Sequences from a Small Read Number of mRNA-Seq Using Hybridomas

**DOI:** 10.1371/journal.pone.0165473

**Published:** 2016-10-27

**Authors:** Yuki Kuniyoshi, Kazumitsu Maehara, Takeshi Iwasaki, Masayasu Hayashi, Yuichiro Semba, Masatoshi Fujita, Yuko Sato, Hiroshi Kimura, Akihito Harada, Yasuyuki Ohkawa

**Affiliations:** 1 Division of Transcriptomics, Medical Institute of Bioregulation, Kyushu University, Fukuoka, Japan; 2 Department of Cellular Biochemistry, Graduate School of Pharmaceutical Sciences, Kyushu University, Fukuoka, Japan; 3 Department of Biological Sciences, Graduate School of Bioscience and Biotechnology, Tokyo Institute of Technology, Yokohama, Japan; Osaka University, JAPAN

## Abstract

Identification of immunoglobulin genes in hybridomas is essential for producing antibodies for research and clinical applications. A couple of methods such as RACE and degenerative PCR have been developed for determination of the *Igh* and *Igl/Igk* coding sequences (CDSs) but it has been difficult to process a number of hybridomas both with accuracy and rapidness. Here, we propose a new strategy for antibody sequence determination by mRNA-seq of hybridomas. We demonstrated that hybridomas highly expressed the *Igh* and *Igl/Igk* genes and that *de novo* transcriptome assembly using mRNA-seq data enabled identification of the CDS of both *Igh* and *Igl/Igk* accurately. Furthermore, we estimated that only 30,000 sequenced reads are required to identify immunoglobulin sequences from four different hybridoma clones. Thus, our approach would facilitate determining variable CDSs drastically.

## Introduction

Hybridomas have been widely accepted as a method for producing large amounts of monoclonal antibodies for research and clinical application [1]. Identification of the amino acid sequence is critical for preserving the characteristics of the antibody, because somatic mutations often occur in the coding region or its regulatory region, resulting in decreased activity of the antibody [2]. Therefore, for the purpose of producing artificial recombinant proteins and filing intellectual properties such as patents, identification of the coding sequences (CDSs) of immunoglobulins is frequently performed to preserve the characteristics of the original antibody.

Antibodies are composed of two subunits; immunoglobulin heavy chain and light chain are each coded by the *Igh* and *Igl/Igk* genes. Both subunits have a constant region and a variable region (V region). The constant region is conserved and codes a crystallizable region (Fc region). The V region contains the V, (D) and J segments, and codes the antigen-binding region, also known as Fab region, while *Igh* has only D segments. These sequences are somatically recombined in pre-B cells, and this recombination plays a key role in antigen specificity and makes it difficult to identify the genomic sequences of immunoglobulin.

A couple of methods have been developed to clone the protein coding sequence of the V region of the *Igh* and *Igl/Igk* genes. The 5´RACE method has been widely used to clone the *Igh* and *Igl/Igk* sequences from hybridomas [3,4]. However, this method requires a large amount of total RNA. The other convenient method is degenerative PCR, which has also been used, but sometimes it causes loss of the original sequence by mis-hybridization of diverse primers [5–8].

Here, we found that the mRNA-seq data of hybridomas contain a substantial amount of reads derived from *Igh* and *Igl/Igk*. *De novo* transcriptome assembly using whole reads obtained by mRNA-seq enabled us to determine the *Igh* and *Igl/Igk* CDSs with only a limited number of reads.

## Materials and Methods

### Cell lines

The hybridoma cell lines used in this study, 4E5 [Hybridoma clone1 (HD1)], 8H3 [Hybridoma clone2 (HD2)], 5A10 [Hybridoma clone3 (HD3)] and 5F11 [Hybridoma clone4 (HD4)], were generated in our laboratory [9–11]. 4E5 clone was established as previously shown [12]. Mouse hybridoma cell lines, 8A2 and 13C7, producing antibodies against histone H3 Lys9 acetylation, were co-established with CMA310 from the same immunized mouse, as previously described [13,14]. Cells were grown in Hybridoma-Serum Free Media (SFM) made from Hybridoma-SFM powder (Gibco), supplemented with 10% FBS, 1.2% penicillin-streptomycin-glutamine (Gibco) and 1 ng/ml IL-6 or in GIT medium (Wako) containing 1 ng/ml IL-6.

### mRNA-seq

Total RNA was extracted from each of the six hybridoma clones (HD1, α-Brg1 antibody, 4E5; HD2, α-Chd2 antibody, 8H3; HD3, α-Chd5 antibody, 5A10; HD4, α-MyoD antibody, 5F11, 8A2, and 13C7) using the AllPrep DNA/RNA Mini Kit (QIAGEN). Library preparation was performed using 1 μg (4E5, 8H3, 5A10 and 5F11) or 3μg (α-Histone H3 lysine 9 acetylatioin (H3K9ac).v2, 8A2 and α-H3K9ac.v3, 13C7) of total RNA and NEBNext Ultra Directional RNA Library Prep Kit (New England Biolabs). mRNA-seq was done with an Illumina HiSeq 1500 for 50 bp (4E5, 8H3, 5A10 and 5F11) or 100bp (8A2 and 13C7) paired-end. More than 40M reads were obtained in each sample (HD1 45M reads, HD2 48M reads, HD3 41M reads, HD4 51M reads). We mainly used the mRNA-seq data of HD1 through HD4 and additionally analyzed 8A2 and 13C7 for the comparative study with Sanger sequencing.

### mRNA-seq data analysis

The reads were mapped against our custom transcriptome reference sequence, which consists of mouse transcripts (generated from UCSC/mm9 refSeq GTF file in Illumina’s igenome reference set), rat transcripts (generated from the NCBI/Rnor5.0 refSeq GTF file in igenome reference set), and rat *Igh* and *Igl/Igk* constant region sequences (obtained from the NCBI nucleotide database; Accession numbers: *Ighg1*: M28670, *Ighg2a*: M28669, *Ighg2b*: M28671, *Ighg2c*: HQ640952, *Iglc1*: M22520, *Iglc2*: M22521, *Igk*: V01241). BWA-MEM was performed to map sequence reads with the parameter: *-t 8 -P -L 10000* (recommended parameter by TIGAR2 [15]). TIGAR2 was run with default settings. The expression level of each gene was quantified as FPKM (fragments per kilobase of exon per million mapped fragments).

### *De novo* transcriptome assembly

Total reads or subsampled reads by fastq-sample (http://homes.cs.washington.edu/~dcjones/fastq-tools) were *de novo* assembled using Trinity. CPU and max_memory parameters were changed according to each read number (e.g., 40M reads:—CPU 8—max_memory 52G; 1M reads—CPU 2—max_memory 12G). We extracted the *Igh* and *Igl/Igk* CDSs by filtering if the contigs contained 20–30 bp of unique sequences of the *Igh* and *Igl/Igk* constant region and had proper length (*Igh* > 1200 bp, *Igl/Igk* > 600 bp). We developed an automation tool to extract immunoglobulin sequences using Trinity output, which is freely available on http://tx.bioreg.kyushu-u.ac.jp/igfinder.

### RT-PCR on the variable region of *Igh* and *Igk*

Each hybridoma RNA was obtained by phenol/chloroform extraction. Reverse transcription was performed using the PrimeScript™ II 1st strand cDNA Synthesis Kit (TaKaRa). PCR was performed using the KOD Plus enzyme (TOYOBO) and a thermal cycler. We used the following thermal protocol: predenature at 94°C for 2 min; 35 cycles of 94°C, 15 s, 55°C, 30 s and 68°C, 20 s; final extension at 68°C for 5 min (HD2-*Igh*, HD3-*Igh*, HD3-*Igk*); predenature at 94°C for 2 min; 35 cycles of 94°C, 15 s, 57°C, 30 s and 68°C, 20 s; final extension at 68°C for 5 min (HD1-*Igh*, HD1-*Igk*, HD2-*Igk*, HD4-*Igk*); predenature at 94°C for 2 min; 35 cycles of 94°C, 15 s, 58°C, 30 s and 68°C, 20 s; final extension at 68°C for 5 min (HD4-*Igh*).

The HD1-*Igh*, HD1-*Igk* and HD4-*Igk* PCR products were purified by gel extraction, to remove non-specific products. Then, all samples were sequenced by Sanger sequencing.

PCR primers:

HD1-*Igh* For: 5’-AAAGCATGTGTGTCTGTGATG-3’ (designed on 5ʹUTR region)

HD2-*Igh* For: 5’-TGAAATCCTCGCAGGAAACTC-3’ (designed on 5´UTR region)

HD3-*Igh* For: 5’-TGATTGCCACAGCCTTCAGT-3’ (designed on 5´UTR region)

HD4-*Igh* For: 5’-CATGAAAACCAGCCTGTCCT-3’ (designed on 5´UTR region)

HD-*Igh* Rev: 5’-AAATAGCCCTTGACCAGGCA-3’ (designed on constant region)

HD1-*Igk* For: 5’-GAAGGTCTTTCTCAGGGCT-3’ (designed on 5´UTR region)

HD2-*Igk* For: 5’-GCTCAGCTGTACTCATGC-3’ (designed on 5´UTR region)

HD3-*Igk* For: 5’-GGTTGGTTGTCATCTTACTGT-3’ (designed on 5´UTR region)

HD4-*Igk* For: 5’-CTTGTCTTGTTGGCTTGAGAT-3’ (designed on 5´UTR region)

HD-*Igk* Rev: 5’-TGATGTCTCTGGGATAGAAGTT-3’ (designed on constant region)

### Data access

mRNA-seq data were submitted to the DDBJ sequence read archive [DRA004264].

## Results

### Identification of the hybridoma *Igh and Igl/Igk* CDSs from mRNA-seq data

**Fig 1:**
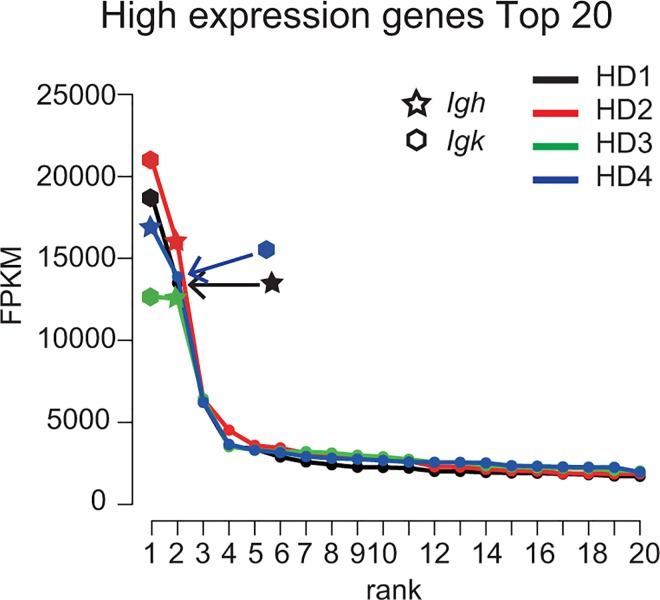
Immunoglobulin gene sequences are highly expressed in all four hybridoma clones. The graph shows 20 top-ranking gene expression levels in four hybridoma clones HD1–4.The X-axis shows the ranking of the gene expression level in all defined transcripts and the Y-axis shows the expression levels in FPKM. The stars and the hexagons indicate the expression level of the *Igh* and *Igk* constant region of the CDS.

We first performed mRNA-seq on four independent hybridoma clones (HD1, α-Brg1 antibody, 4E5; HD2, α-Chd2 antibody, 8H3 [9]; HD3, α-Chd5 antibody, 5A10 [10]; HD4, α-MyoD antibody, 5F11[11]) that were established as fusion cells of rat B lymphocytes and mouse myeloma cell line SP2 (paired-end 50 bp reads). Then, we comprehensively quantified each transcriptome expression level by BWA-TIGAR2 [15] and ordered them according to expression levels (Fig 1). The data showed that the CDSs of the *Igh* and *Igl/Igk* constant region were ranked as the highest expressed transcripts (FPKM > 10000) in all four hybridoma lines (Fig 1). This suggests that the mRNAseq data of hybridomas contained enough number of reads to reconstruct the CDSs of *Igh* and *Igl/Igk* [16].

**Fig 2:**
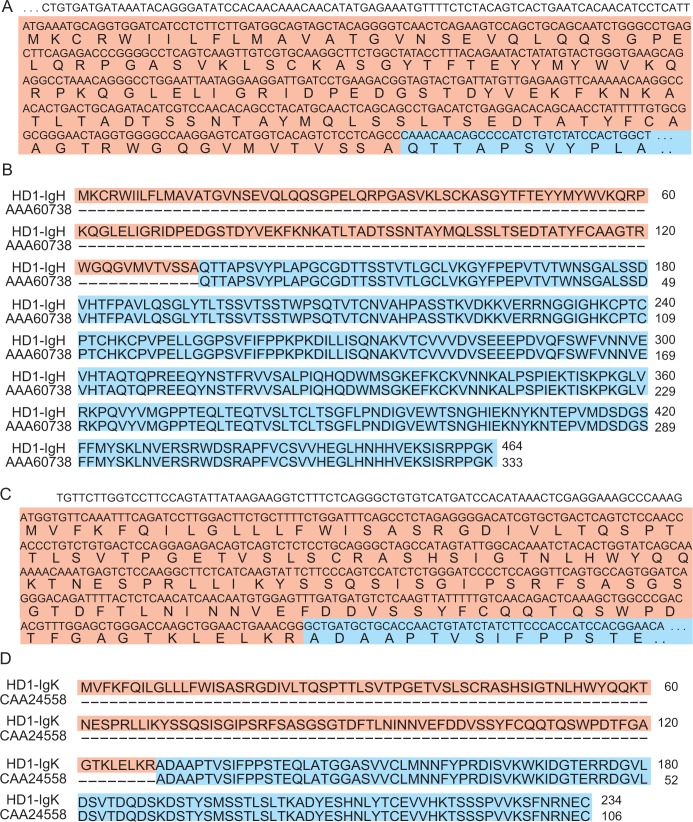
Identified immunoglobulin nucleotide and protein sequences of HD1. (A) The partial nucleotide sequence of *Igh* and the corresponding amino acid sequence. (B) Sequence alignment of HD1 IgH protein (top lane) and the known rat IgH constant region (bottom lane). The *Igh* CDS of HD1 codes the amino acid sequence of the Ig gamma-2B chain C region. (C) The partial nucleotide sequence of *Igk* and the amino acid sequence. The *Igh* CDS and *Igk* CDS were obtained after *de novo* assembly of HD1 mRNA-seq data and filtering contigs. (D) Alignment of the IgK protein sequence of HD1 and the known rat IgK constant region. The *Igk* CDS of HD1 codes the amino acids of the Ig kappa chain C region. White, red and blue backgrounds show the UTR region, V region and C region, respectively.

Next, we attempted to reconstruct the *Igh* and *Igl/Igk* CDSs by *de novo* transcriptome assembly of the hybridoma mRNA-seq data. First, the mRNA-seq data obtained from hybridoma clone 1 (HD1) were simply assembled with Trinity which reconstructs a full-length transcriptome from RNA-seq data without a genome [16]) without filtering reads (45,406,048 reads), obtaining 58,822 contigs. We further extracted the *Igh* CDS by filtering if the contigs contained 20–30-bp unique sequences of the *Igh* constant region, which can uniquely determine each gene (rat-*Ighg1*: TGTGCCCAGAAACTGTGGAG, rat-*Ighg2a*: GCCAAGGGAATGCAATCCTTG, rat-*Ighg2b*: CAAACAACAGCCCCATCTGTCTAT, rat-*Ighg2c*: AGAACAACAGCCCCATCTGTCTA). As the full length of IgH has more than 400 amino acids (aa) [17], a 1395-bp sequence was obtained as the *Igh* CDS containing the unique 24-bp sequence of *Ighg2b* after filtering if the contig has more than 1200 bp. The V region and partial constant region of the obtained *Igh* CDS were confirmed to be identical to the sequence obtained by Sanger sequencing of reverse-transcribed PCR (RT-PCR) products which were amplified with primer sets designed based on the 5'UTR region and constant region. Alignment of the obtained IgH protein sequence and the known rat IgH constant region (AAA60738) confirmed matching of the full length of amino acid sequence of the constant region in the known IgG2b and the 133–464 amino acids identified in the *Igh* CDS (Fig 2B). We also extracted the *Igl/Igk* CDS from the contigs by filtering if it contains 20–30-bp unique sequences of the known *Igl/Igk* constant region (rat-*Igl1*: CAACCCAAGGCTACGCCCTC, rat-*Igl2*: CAGCCCAAGTCCACTCCCAC, rat-*Igk*: ACCAACTGTATCTATCTTCCCACCATCCAC). As the full length of IgK has more than 200 aa [17], a 705-bp sequence was obtained as the *Igk* CDS after filtering if the contig has more than 600 bp (Fig 2C). We also confirmed the V region and partial constant region of the obtained *Igk* CDS with Sanger sequencing following RT-PCR. We demonstrated matching of the amino acid sequence of the constant region in known IgK (CAA24558) and the amino acids of the identified *Igk* CDS (Fig 2D). We also identified the *Igh* and *Igl/Igk* CDSs of HD2 (*Ighg2a /Igk*), HD3 (*Ighg2a /Igk*) and HD4 (*Ighg2a /Igk*) (data not shown). Then, these identified antibody isotypes corresponded to the results of ELISA-format isotyping assay. Mouse *Igh* and *Igk* CDSs from mouse hybridoma clones (8A2, 13C7) were also identified and their amino acids sequences were identical to coding sequence determined by PCR cloning shown in [7,13] except the regions coded on sequences on degenerative sequences (S1 Fig). Mouse *Igh* and *Igl/Igk* transcripts was extracted by unique sequences of the mouse *Igh* and *Igl/Igk* constant region (mouse-*Ighg1*: CCAAAACGACACCCCCATCT, mouse-*Ighg2a*: GTGTGTGGAGATACAACTGGCT, mouse-*Ighg2b*: CCAAAACAACACCCCCATCAG, mouse-*Ighg2c*: GTGTGGAGGTACAACTGGCTCCT, mouse-*Ighg3*: CTACAACAACAGCCCCATCTG, mouse-*Igl1*: GCCAGCCCAAGTCTTCGCCAT, mouse-*Igl2*: GTCAGCCCAAGTCCACTCCCACTC, mouse-*Igl3*: GTCAGCCCAAGTCCACTCCCACAC, mouse-*Igl4*: GCCAACCCAAGGCTACACCCTCAG, mouse-*Igk*: GGGCTGATGCTGCACCAACTG). These results indicated that simple *de novo* assembly using hybridoma mRNA-seq data was beneficial for identifying both the *Igh* and *Igl/Igk* genes.

### Optimization of *de novo* transcriptome assembly for identifying immunoglobulin sequences

**Fig 3:**
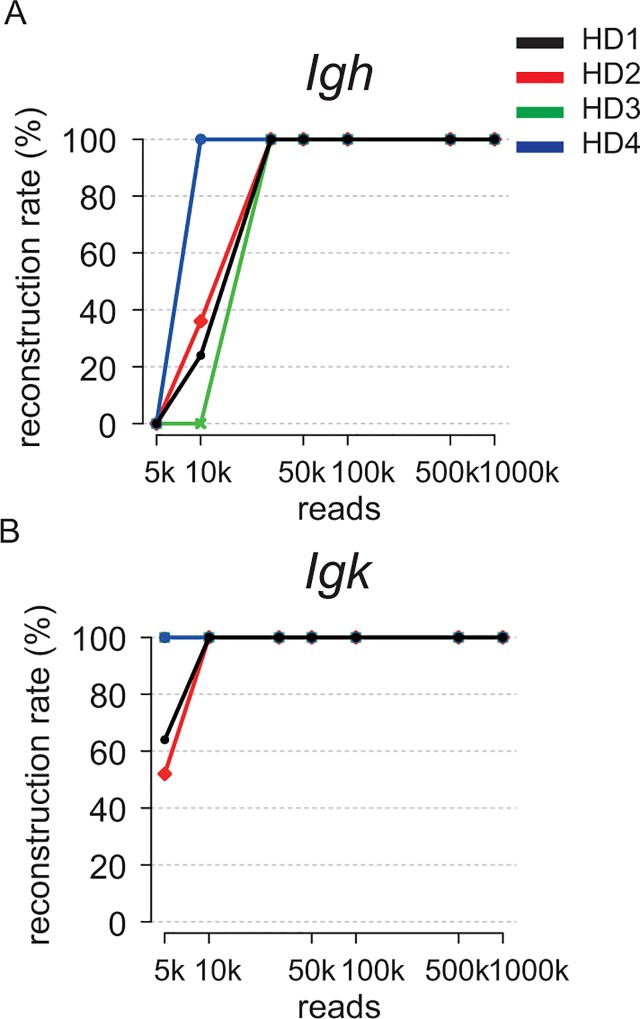
Required number of reads to identify immunoglobulin gene sequences was only 10–30k. The number of reads used for *de novo* assembly and the success rate (%) of obtaining complete *Igh* CDS (A) and *Igk* CDS (B). Immunoglobulin sequences from four hybridomas were assembled with 30k reads. The random selection of reads and *de novo* assembly were performed 25 times for each number of subsampled reads.

**Fig 4:**
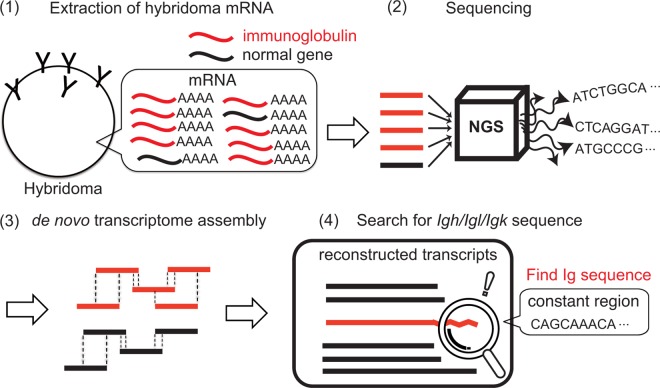
Schematic drawing of the immunoglobulin gene sequences identification using a low read number of hybridoma mRNA-seq data. First, mRNA-seq of hybridoma is performed (step 1, 2). Next, mRNA-seq data is *de novo* assembled (step 3). Finally, promising immunoglobulin gene sequences are identified by filtering contigs according to the requirements for coding valid immunoglobulin protein sequences (step 4).

We further attempted to optimize the strategy for identifying the CDSs of the *Igh* and *Igl/Igk* genes using hybridoma mRNA-seq data. First, to estimate the required number of reads for identifying an immunoglobulin gene by our method, we randomly subsampled 5k, 10k, 30k, 50k, 100k, 500k and 1M reads from the total reads in the mRNA-seq data of four different hybridomas (HD1, HD2, HD3 and HD4). Then, we repeated the *de novo* assembly 25 times using the randomly selected reads. We defined the *Igh* and *Igk* CDSs identified by the *de novo* assembly using the total reads (such as in Fig 2A and 2C) as correct sequences, and then calculated the success rate of obtaining complete CDSs (Fig 3A and 3B). The *Igh* and *Igk* CDSs of all four clones were perfectly identified with > 30k reads (Fig 3A) and > 10k (Fig 3B) reads, respectively. This result confirmed that our method successfully identified immunoglobulin sequences with limited reads from mRNA-seq data (Fig 4).

We implemented our immunoglobulin sequence identification strategy with a python script named *igfinder* available at our website (http://tx.bioreg.kyushu-u.ac.jp/igfinder).

## Discussion

Here, we propose a rapid and accurate method for identifying the CDSs of *Igh* and *Igl/Igk* by *de novo* transcriptome assembly. Our method requires limited reads of mRNA-seq, because hybridomas highly express *Igh* and *Igl/Igk* transcripts. Our approach would be beneficial for rapid and cost-effective cloning of *Igh* and *Igl/Igk* CDSs.

Conventionally, PCR, 5'RACE and SMARTer RACE (Clontech) have been used with degenerative primers for the determination of antibody sequences (S1 Table). 5´RACE has been widely used to identify *Igh* and *Igl/Igk* CDSs from hybridomas; however, it is time consuming and requires a large amount of total RNA. SMARTer RACE, which is a refinement on 5'RACE, requires only a small amount of RNA; however, SMARTer RACE and 5’RACE occasionally extract pseudo-sequences caused by annealing or mis-annealing of primers to the myeloma cell-derived *Igh* or *Igl/Igk* sequences in the hybridoma [3]. Therefore, several clones’ identified sequences should be confirmed by other approaches such as Sanger sequencing of RT-PCR products. Our method avoided this procedure, because the *Igh* and *Igl/Igk* sequences were selected by filtering sequences based on the CDS length. We also surmise, on account of the remarkably high expression of Ig genes, that our method can work with as low as ~0.1μg of total RNA, which is the minimal requirement for the library prep kit used in this study.

Our method depends on the quantity of *Igh* and *Igl/Igk* transcripts in each hybridoma. Therefore, hybridomas that express antibodies with a low level of *Igh* and *Igl/Igk* may not have enough transcripts to be assembled. In this case, increasing the read number for *de novo* assembly could be beneficial for identifying the *Igh* and *Igl/Igk* CDSs [16].

Another advantage of our method is the ability to identify full-length *Igh* and *Igl/Igk*, while other methods only identify the V region of immunoglobulin genes. Therefore, our method enables identification of the antibody isotypes and subclasses (e.g., rat- *Ighg1*, *Ighg2a*, *Ighg2b*, *Ighg2c*). We hope to extend our method in our future work for the detection of minor variants of antibody genes caused by somatic mutations, e.g., in clinical samples of myeloma or lymphoma cells.

A recent study has demonstrated that mouse plasma cells highly express immunoglobulin genes [18]. Hybridomas also highly express immunoglobulin genes, which are derived from a B cell fused with a myeloma cell. Our data suggest that it is possible to identify *Igh* and *Igl/Igk* CDSs, even from intact B cells or plasma cells, even at the single cell level.

## Supporting Information

S1 Fig Alignment of the Immunoglobulin amino acid sequences identified from mouse hybridoma clones. (EPS)

S1 Table Comparative table of methods for determining Ig sequences and their features. (PDF)
