# Supplementary material for: Identification of Immunoglobulin Gene Sequences from a Small Read Number of mRNA-Seq Using Hybridomas
Source: PLoS One. 2016 Oct 27;11(10):e0165473. doi: 10.1371/journal.pone.0165473 (PMC5082856; doi:10.1371/journal.pone.0165473)
Supplement: S1 Table — (PDF) [file pone.0165473.s002.pdf]

S1 Table. Comparative table of methods for determining Ig sequences and their features

Method	Subclass Identification	Sample Analysis Throughput	Cost/Sample	Procedure (days)	Total Duration[7]
Degenerative PCR	by degenerative primers for constant region	Low	~$20[1]+~$200[2]	1. RNA-extraction (.5)<br>2. RT-PCR and cloning (2)<br>3. Sanger (2)	4-14 days
5'RACE & Sanger	by subclass specific primers for constant region	Low	~$100[3]+$200[2]	1. RNA-extraction and RT cDNA synthesis (.5)<br>2. RACE PCR and cloning (2)<br>3. Sanger (2)	4-14 days
SMARTer 5'RACE & Sanger	by subclass specific primers for constant region	Low	~$100[4]+$200[2]	1. RNA-extraction (.5)<br>2. SMARTer cDNA synthesis (.5)<br>3. RACE PCR and cloning (2)<br>4. Sanger (2)	4-14 days
RNA-Seq & de novo assembly	in silico	High<br>192 samples	~$120-200[5] + ~$30[6]	1. RNA-extraction and library prep (2)<br>2. RNA-Seq (2)	4 days

~ : approximately

*1,3,4 indicate sequencing sample prep costs. *2 indicates Sanger sequencing costs for 10 heavy and 10 light chain clones

*3 SuperScriptIII (Thermo Fisher Scientific), 5' RACE System for Rapid Amplification of cDNA Ends, version 2.0 (Thermo Fisher Scientific), TAKARA primer STAR (Takara bio, Shiga, JAPAN), DNA Ligation Kit <Mighty Mix> (TAKARA Bio), and QIAquick Gel Extraction Kit (Qiagen, hilden Germany).

*4 SMARTer® RACE 5'/3' Kit (TAKARA Bio), TAKARA primer STAR (Takara bio, Shiga, JAPAN), DNA Ligation Kit <Mighty Mix> (TAKARA Bio), and QIAquick Gel Extraction Kit (Qiagen, hilden Germany).

*5 indicates library prep cost http://healthcare.utah.edu/huntsmancancerinstitute/research/shared-resources/center-managed/high-throughput-genomics-site/pricing/illumina-hiseq-pricing.php and http://www.rockefeller.edu/genomics/pricing

*6 Hiseq sequencing costs (rapid run mode, paired-end, 2x50bp sequencing lane [96 samples, one lane], per lane)

*7 With an increased number of pseudo sequences, the later part of the procedure has to be repeated. The total duration was calculated with the assumption of a few repetitions.
